# Utilizing Focus Groups to Inform the Development of the Financial Empowerment Scale

**DOI:** 10.1093/geroni/igaf122.229

**Published:** 2025-12-31

**Authors:** Carson De Fries, Riley Coyne, Pilar Ingle, Ingrid Backes, Briana Kohlbrenner, Shannon O’Gara-Standiford, Asia Cutforth, Eric Chess

**Affiliations:** University of Utah, Salt Lake City, Utah, United States; University of Denver, Denver, Colorado, United States; University of Denver, Denver, Colorado, United States; University of Denver, Denver, Colorado, United States; University of Denver, Denver, Colorado, United States; University of Denver, Denver, Colorado, United States; University of Denver, Denver, Colorado, United States; University of Denver, Denver, Colorado, United States

## Abstract

Financial fraud and exploitation (FFE) are associated with billions of dollars lost each year and have significant adverse effects on older adults. Multiple domains have been identified that impact the risk of FFE among older adults including individual behaviors, personality traits, social factors, financial literacy, and cognitive health. However, no accessible tool comprehensively assesses these factors and the ways they intersect to better identify potential risk of FFE. This study is part of a larger ongoing project to develop a scale assessing financial wellbeing among older adults and aims to: (1) determine key domains associated with risk for FFE, (2) inform the development of the Financial Empowerment Scale (FES), and (3) assess the need for and applicability of the FES from different professional viewpoints. To achieve these aims, semi-structured focus groups were conducted with experts in the field. Thirty-six individuals across six focus groups participated, representing financial institutions, academia, and aging and social services. Data were analyzed through an iterative, mixed inductive-deductive content analysis approach. Five domains were identified as core components influencing risk factors for FFE: Cognitive Health, Financial Wellbeing, Social Health, Individual Behaviors, and Health and Environment. Focus group participants also expressed the need for a scale such as the FES to support older adults and professionals in the field. Findings from this study expand on the literature surrounding FFE among older adults and support the need for FES development. Findings around domains will be applied to item generation in the development of the FES.

